# Fungal Community Composition and Its Relationship with Volatile Compounds during Spontaneous Fermentation of Cabernet Sauvignon from Two Chinese Wine-Growing Regions

**DOI:** 10.3390/foods13010106

**Published:** 2023-12-28

**Authors:** Jie Gao, Huiying Geng, Ruru Chai, Tianyang Wu, Weidong Huang, Yilin You, Jicheng Zhan

**Affiliations:** Beijing Key Laboratory of Viticulture and Enology, College of Food Science and Nutritional Engineering, China Agricultural University, Tsinghua East Road 17, Haidian District, Beijing 100083, China; bs20213060545@cau.edu.cn (J.G.); genggengyuhuai@cau.edu.cn (H.G.); chai17860730309@126.com (R.C.); tianyangwu@cau.edu.cn (T.W.); huanggwd@263.net (W.H.); yilinyou@cau.edu.cn (Y.Y.)

**Keywords:** cabernet sauvignon, spontaneous fermentation, wine-growing region, fungal communities, volatile compound

## Abstract

The microbial community structure associated with wine in a wine-growing region is shaped by diverse ecological factors within that region, profoundly impacting the wine flavor. In wine fermentation, fungi contribute more sensory-active biochemical compounds than bacteria. In this study, we employed amplicon sequencing to measure samples from the spontaneous fermentation process of cabernet sauvignon wines from two wine-growing regions in China to study the diversity and structural evolution of fungi during spontaneous fermentation and analyze the correlation between fungi and volatile compounds. The results showed significant differences in fungal community structure and diversity in cabernet sauvignon musts from different geographical origins, and these differences affected the flavor quality of the wines. As alcoholic fermentation progressed, *Saccharomyces* became the dominant fungal genus and reshaped the fungal community structure, and the diversity of the fungal community decreased. However, the fungal communities of each wine-growing region remained distinct throughout the fermentation process. Furthermore, the correlation between the fungal community and volatile compounds indicated that wine is a product of fermentation involving multiple fungal genera, and the flavor is influenced by a variety of fungi. Our study enhances the comprehension of fungal communities in Chinese wine-growing regions, explaining the regulatory role of wine-related fungal microorganisms in wine flavor.

## 1. Introduction

Wine, as an economic and cultural commodity, is highly appreciated by consumers for its diverse style characteristics. The regional differences in the quality and style of wine influenced by terroir have an impact on consumer preferences [1]. Terroir is a very important concept in viticulture and winemaking, covering factors such as climate, soil conditions, cultivation practices, and human activities during the wine grape growth process. It links the sensory characteristics of the wine to the growing environment of the wine grape [2]. With the advancement of gene sequencing technology, the biogeography of grape-related microorganisms has been confirmed to be non-randomly distributed. Microbial biogeographical patterns are influenced by factors such as the geographical location of the wine-growing regions, climatic conditions, soil properties, topographic features, grape varieties, and viticultural practices [3,4]. Researchers have introduced microorganisms into the concept of terroir and proposed the term “microbial terroir”, listing microorganisms as one of the important contributors to the flavor profiles of wine-growing regions, demonstrating that microorganisms also greatly affect the health of grapes and the flavor quality of wines [5].

Grapevines and their surrounding environment contain a variety of microorganisms, including yeasts, filamentous fungi, and bacteria. These microorganisms can disperse from the soil in the vineyard, the vegetation surrounding grapevines, and nearby forests through the air, precipitation, or animals such as drosophila and honeybees [6,7,8,9]. These microorganisms have different physiological characteristics and greatly regulate the growth and health of grapes through interactions such as symbiosis, mutualism, or pathogenesis [10]. Wine fermentation is a complex mixed-strain fermentation system involving a large amount of biotransformation by fungi and bacteria. The aromatic compounds in wine are associated with the microbial community distribution patterns on wine grapes [11,12,13]. During the fermentation process, microorganisms, along with grapes or juice, are involved in winemaking, including numerous microbes such as *Saccharomyces cerevisiae* that participate in the fermentation of wine. They convert sugar in grapes into alcohol and simultaneously produce numerous secondary metabolites, including but not limited to higher alcohols, esters, volatile fatty acids, and other volatile compounds that affect the aromatic quality of the wine, forming its vinous character [14]. The types of microorganisms involved in wine fermentation and the interactions between them can affect the final physicochemical properties and flavor profiles of the wine [15,16]. For example, Lactobacillus can release free aromatic compounds by secreting glycosidases and can change the concentration of ester compounds in wine through the secretion of esterases, thus changing its overall flavor [17]. Compared with bacteria, the regional characteristics of wine are more closely associated with fungal ecology [12,18].

Currently, China is the country with the third largest area under vines in the world after Spain and France, with 785 kha of vineyards [19]. Among them, the planting area of wine grapes is approximately 163.2 kha. These are widely distributed across 179 counties of China, spanning from latitude 24° N to 47° N and longitude 76° E to 132° E [20]. Variations in geography and topography result in diverse climate characteristics among Chinese wine-growing regions, as well as distinct microbial species resources [21]. However, current research on microbial communities in wine-growing regions is still in its early stage, mainly focusing on characterizing the microbial structure of individual regions [22,23,24]. The differences in the biogeographical distribution patterns of microorganisms between Chinese wine-growing regions and their impact on wine flavor require further study.

This study aimed to explore the influence of geographical origin on the structure of fungal communities during spontaneous fermentation and the flavor of cabernet sauvignon wines from Yinchuan, Ningxia and Fangshan, Beijing. To achieve this, we used amplicon sequencing technology to characterize the fungal communities in cabernet sauvignon musts and spontaneous fermentation samples from two wine-growing regions 880 km apart, each represented by three wineries, and analyzed the composition and dynamic changes of fungi during the fermentation process. High-performance liquid chromatography (HPLC) was used to measure the physicochemical properties of the musts and wines, and headspace solid-phase microextraction (HS-SPME) followed by gas chromatography–mass spectrometry (GC-MS) was employed to perform the untargeted volatile profiling of metabolites in the samples. Furthermore, we elucidated the associations between fungal microbiota and aromatic compounds in the wines to reveal the potential impact of regional fungal structures on their aromas.

## 2. Materials and Methods

### 2.1. Winemaking and Sampling

In 2022, three wineries were selected from both Yinchuan, Ningxia (designated as NC, with wineries labeled HJZ, JX, and ZHYS) and Fangshan, Beijing (designated as BC, with wineries labeled BLB, XL, and ZW) in China to collect cabernet sauvignon grape samples for this study (Supplementary Figure S1). The distance between the two regions is approximately 880 km. The grapes were destemmed, crushed, and pressed by mechanical equipment at the wineries and then transported back to the laboratory at low temperatures. The physicochemical parameters of cabernet sauvignon musts/wines are listed in Table 1. The cabernet sauvignon musts were loaded in triplicate into 5 L fermentation tanks (working volume: 4 L) and cold-soaked at 4 °C for 48 h. After the temperature of the grape musts were raised to 20 °C, spontaneous fermentation was conducted without the inoculation of commercial *S. cerevisiae*. During the fermentation, the temperature was controlled at 20 °C, and the samples were stirred twice a day. Samples were collected in triplicate in sterile centrifuge tubes at five time points: cabernet sauvignon must (Must, crushed cabernet sauvignon must before fermentation); at the start of fermentation (S2, approximately 10 g/L of sugar fermented); in the early stage of fermentation (S3, approximately 25% of the sugar fermented); in the late stage of fermentation (S4, about 75% of the sugar fermented); and at the end of fermentation (S5, total sugar content less than 4 g/L). Each sample was divided into two sub-samples after sampling; one was stored at −20 °C for the determination of physicochemical parameters and volatile compounds, and the other was stored at −80 °C for DNA extraction and amplicon sequencing.

### 2.2. Cabernet Sauvignon Inoculated Fermentation

After cold soaking at 4 °C, dimethyl ecarbonate (DMDC) (Aladdin Chemistry Co., Ltd., Shanghai, China) was added to the grape juice at a concentration of 400 μL/L, which was then incubated overnight at 25 °C with agitation at 100 rpm for chemical sterilization, according to the method described by Pinu et al. [25]. Microorganisms on the surface of grape berries were collected following Chen’s method with modifications [26]. Using the five-point sampling method, 400 g of cabernet sauvignon grapes were randomly sampled from Bolongbao Chateau in Beijing (B) and Yuanshi Chateau in Ningxia (Z). Cabernet sauvignon grape berries, weighing 200 g, were soaked in 500 mL of sterile 10X PBS solution and vortexed for 15 min to elute the microorganisms from the grape surface. The eluted solutions were centrifuged at 5000 rpm for 10 min to enrich the microorganisms. The precipitated microorganisms were cultured in 100 mL of chemically sterilized grape juice at 20 °C for 24 h and then inoculated into 2 L of sterilized grape juice at an inoculum volume of 2% for inoculated fermentation. The Inoculated fermentation was conducted at 20 °C with three parallels in each group. The sampling time points were the same as those for the spontaneous fermentation, and the samples were stored at −20 °C for the determination of volatile compounds. The abbreviations used for the inoculated fermentation samples and their descriptions are displayed in Table 2.

### 2.3. DNA Extraction and Sequencing

Genomic DNA amplification and sequencing were conducted by Majorbio Bio-pharm Technology Co., Ltd. (Shanghai, China). The extraction and sequencing of DNA from the samples were carried out using the method previously described in [27].

We used fastp software (https://github.com/OpenGene/fastp, accessed on 3 May 2023, version 0.19.6) to control the quality of paired-end raw sequences and filter low-quality sequences. According to the sequence overlap relationships, FLASH (http://www.cbcb.umd.edu/software/flash, accessed on 3 May 2023, version 1.2.7) software was used to splice the paired ends. The DADA2 approach was used to perform denoising on the sequences after quality control and splicing, and we removed as many PCR amplification errors or sequencing errors in the data as possible to obtain the true sequence information within the sample. The sequences after denoising were called amplicon sequence variants (ASVs). Based on the Unite 8.0 database, the taxonomic classification of ASVs was conducted using the naive Bayes classifier in Qiime2. To avoid or reduce the influence of varying sequencing depths on the analysis results, the lowest sequencing depth in the sample was used to normalize the sequence data of all samples, ensuring that each sample had the same sequencing depth. The raw data were uploaded to the Sequence Read Archive (SRA) database of the National Center for Biotechnology Information (NCBI) under Bioproject PRJNA1054229.

### 2.4. Physicochemical Parameter Analysis

The basic physicochemical parameters of cabernet sauvignon musts and wines were quantified by HPLC using the method previously described in [27].

### 2.5. Analysis of Volatile Compounds

According to the method described by Gao [27], the concentrations of volatile compounds in samples from five stages of spontaneous fermentation and in wine samples at the end of inoculated fermentation were determined using headspace solid-phase microextraction (HS-SPME) followed by gas chromatography–mass spectrometry (GC–MS).

### 2.6. Statistical Analysis

Three replicates of all samples were used for analysis, and the results are presented in the form of mean ± standard deviation. The α-diversity index of the microbial communities was calculated using the Mothur software package (version 1.30). Principal coordinate analysis (pCoA) based on the unweighted UniFrac distance was performed to evaluate the distribution patterns of samples, and an analysis of similarities (ANOSIM) was used to determine the significance of differences in microbial community structure between sample groups. Linear discriminant analysis (LDA) effect size (lEfSe) was calculated to analyze the significant taxonomic differences in the fungi between the musts from the two grape-growing regions. Using the table containing only ASVs with a relative abundance >0.01%, the factorial Kruskal–Wallis sum-rank test was applied to identify taxonomic genera with significant abundance differences between regions (comparison strategy: all-against-all comparisons), and logarithmic LDA scores (threshold = 2.0) were used to assess their effect size. The Wilcoxon rank-sum test was used to identify taxonomic genera with significant abundance differences between the two regions in different fermentation stages. Student’s *t*-test and one-way analysis of variance (ANOVA, significance level 0.05) based on Tukey’s test were completed using SPSS 25.0 software (SPSS Inc., Chicago, IL, USA). The normality and homogeneity of variances were tested using the Shapiro–Wilk test and Levene’s test. Principal component analysis (PCA) to display the distribution patterns of wine samples was performed using Origin 2019b software (OriginLab, Northampton, MA, USA), and permutational multivariate analysis of variance (PERMANOVA) was conducted based on the Euclidean distance to test the significance of differences between the sample groups. Orthogonal partial least-squares–discriminant analysis (OPLS-DA) was conducted using SIMCA software (version 14.1, UMETRICS, Umea, Sweden) and combined with variable importance in projection (VIP) scores greater than 1 to find differential metabolites between groups. The correlation between fungal microorganisms and volatile compounds based on Spearman’s correlation coefficient was visualized using R (version 4.1.2).

## 3. Results

### 3.1. Fungal Communities Varied by Wine-Growing Region

**Figure 1:**
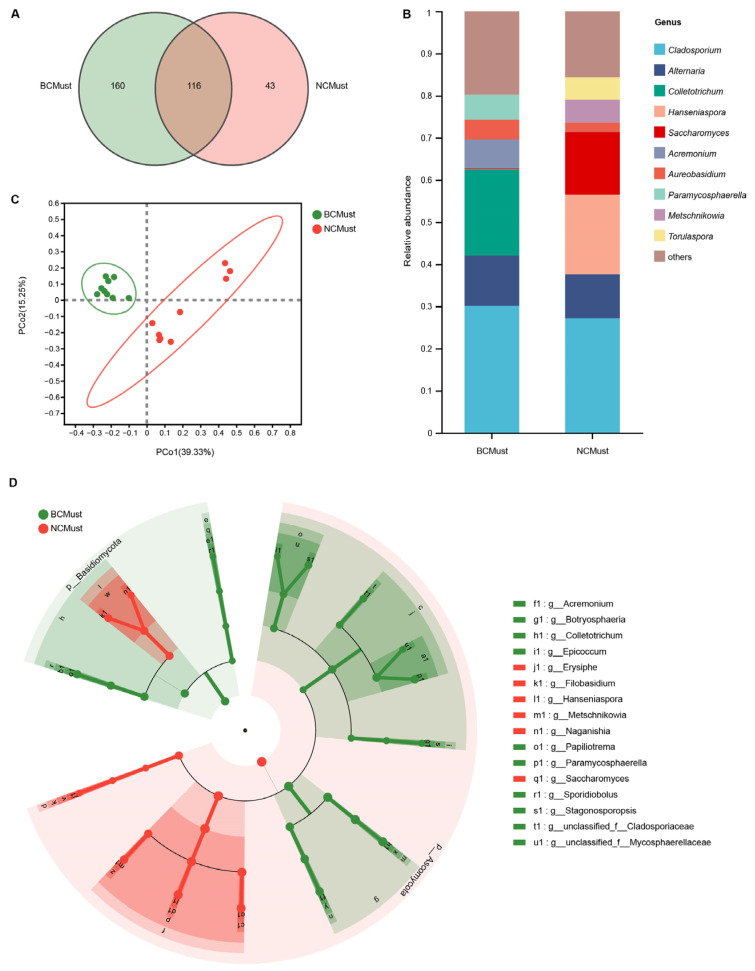
The fungal communities in the cabernet sauvignon musts varied depending on their geographical origin. (**A**) Venn diagram of fungal genera in samples from the two wine-growing regions. (**B**) Fungal community composition at the genus level (top 10 shown). (**C**) pCoA based on unweighted UniFrac distances between samples from the two regions. (**D**) Linear discriminant analysis (LDA) effect size (lEfSe) taxonomic cladogram for fungal biomarkers associated with geographical origin (LDA score > 2.00, *p* < 0.05).

To analyze the variations in the fungal communities throughout the fermentation of cabernet sauvignon wines from the two wine-growing regions, a total of 90 samples covering six wineries were collected during five fermentation periods. A total of 5,862,936 ITS sequences were generated from all samples, which were clustered into 2228 ASVs, and 7 phyla, 26 classes, 63 orders, 168 families, 395 genera, and 740 species were identified. Among them, Ascomycota was the most abundant phylum in cabernet sauvignon musts, accounting for 93.18% of all sequences, followed by Basidiomycota with a relative abundance of 6.69%. The combined relative abundance of Chytridiomycota and Mortierellomycota in cabernet sauvignon must was less than 0.01%. At the genus level, a total of 319 genera of fungi were identified in the cabernet sauvignon musts from the two wine-growing regions. Of these, 116 genera were common to both regions, 160 genera were only present in BC, and 43 genera were unique to NC (Figure 1A). The genus with the highest relative abundance in the musts was *Cladosporium*, accounting for 28.63% of all sequences. Together with *Alternaria*, *Colletotrichum*, *Hanseniaspora*, *Saccharomyces*, *Acremonium*, *Aureobasidium*, *Paramycosphaerella*, *Metschnikowia*, and *Torulaspora*, they collectively constituted the top ten fungal genera by relative abundance in the musts (Figure 1B).

The pCoA of the unweighted UniFrac distance was used to visualize the distribution pattern of fungal communities in the two wine-growing regions (based on a 95% confidence interval), with the first two principal coordinate (PC) axes explaining 54.58% of the total variance (Figure 1C). The fungal communities in the cabernet sauvignon musts from the two wine-growing regions were significantly separated, confirming that there were significant geographical differences in the composition of the fungal community in the musts (ANOSIM, R = 0.7459, *p* = 0.001). The wine-growing regions could be distinguished based on the fungal microbiota in the cabernet sauvignon musts. The lEfSe analysis further confirmed that there was a significant association between the abundance of fungi at the genus level and the geographical origin of the wineries (Figure 1D). The lEfSe results revealed that there were 16 fungal taxa exhibiting significant differences between the two wine-growing regions. Among them, six fungal genera, *Erysiphe*, *Filobasidium*, *Hanseniaspora*, *Metschnikowia*, *Naganishia*, and *Saccharomyces*, were significantly enriched in NC. Conversely, *Acremonium*, *Botryosphaeria*, *Colletotrichum*, *Epicoccum*, *Papiliotrema*, *Paramycosphaerella*, *Sporidiobolus*, *Stagonosporopsis*, and two unclassified fungal genera were significantly more abundant in BC.

### 3.2. Dynamics of Fungal Communities during Spontaneous Fermentation

**Figure 2:**
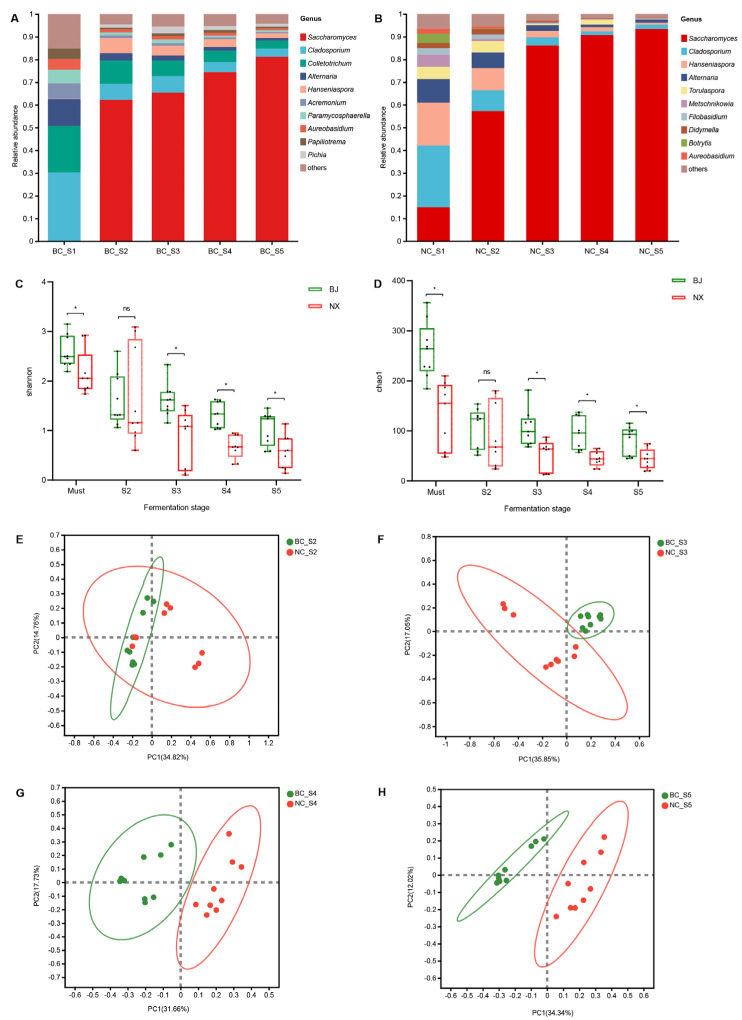
Composition and diversity of fungal communities during spontaneous fermentation. Relative abundance changes of fungal genera in samples from BC (**A**) and NC (**B**) (showing the top 10 genera). Changes in fungal diversity during spontaneous fermentation of cabernet sauvignon in the two wine-growing regions (**C**,**D**). pCoA of fungal communities between the two wine-growing regions at different fermentation stages (**E**–**H**). Note: *p*-values are indicated with asterisks (*). * 0.01 < *p* < 0.05, ns indicates no significance.

The main fungal genera (top 10 abundant taxa) in the fermentation samples from the two wine-growing regions were analyzed, revealing the dynamic changes in the fungi during the fermentation process (Figure 2A,B). The relative abundance of the major fungal genera changed significantly as spontaneous fermentation progressed. In the musts, filamentous fungi and non-*Saccharomyces* yeasts accounted for the majority of fungal community abundance, with *Cladosporium* being the most abundant genus in samples from both wine-growing regions. After the start of fermentation, *Saccharomyces* rapidly proliferated and assumed a dominant role in alcoholic fermentation. In NC, the relative abundance of *Saccharomyces* in the fungal community increased from 14.92% (Must) to 93.41% (Stage 5). The relative abundance of *Saccharomyces* in the fungal community increased from 0.18% (Must) to 81.14% (Stage 5) in BC. From the early stage of fermentation (Stage 3), the relative abundance of *Saccharomyces* in NC was significantly higher than that in BC (Supplementary Figure S2). The relative abundance of other fungal genera decreased as fermentation progressed. It is worth noting that after fermentation began, the relative abundance of *Pichia* in BC was always significantly higher than that in NC (Supplementary Figure S2).

Alpha diversity is primarily used to study the diversity of communities within samples, serving as a comprehensive metric that reflects factors such as the richness and evenness of the community within the sample [28]. The α-diversity of fungal communities in fermentation samples from the two regions was compared through the Shannon index and the Chao1 estimator (Figure 2C,D). The diversity of fungal communities was significantly reduced during spontaneous fermentation (*p* < 0.05). Except for the fermentation initiation stage (Stage 2), the fungal community diversity observed in the BC samples consistently surpassed that of the NC (Figure 2C,D). The pCoA showed that the fungal microbiota of the two wine-growing regions were clearly separated at different fermentation stages (Figure 2E–H). The significant geographical differences in fungal genera between the two wine-growing regions during fermentation suggested that geographical origin can be determined based on microbial community characteristics (Supplementary Table S1).

### 3.3. Aroma Characteristics of Wines Varied by Wine-Growing Region

**Figure 3:**
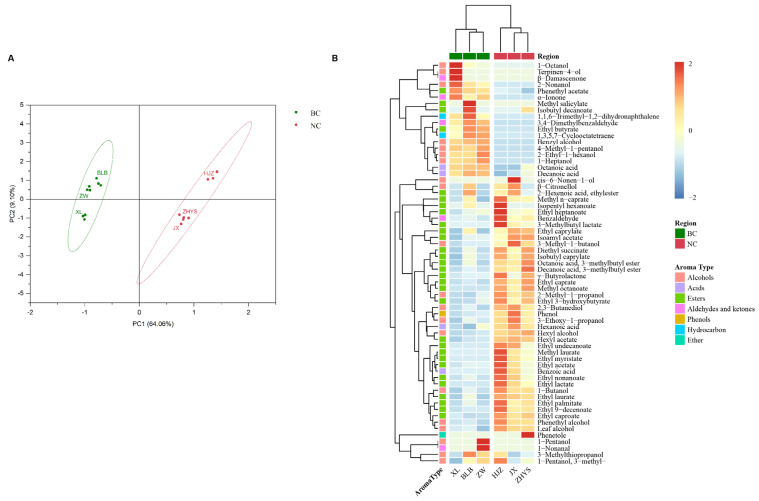
The volatile compounds in wines exhibited regional differences. (**A**) PCA score plot of volatile compounds in wines from two wine-growing regions. (**B**) Clustered heatmap of volatile compounds in wines from two wine-growing regions.

Wine aroma is complex, primarily dependent on volatile and semi-volatile compounds derived from the grape berries and formed during fermentation, including aldehydes, ketones, terpenes, higher alcohols, volatile fatty acids, and esters [29]. These compounds play a crucial role in shaping the overall and sensory characteristics of wine. The volatile compounds in the cabernet sauvignon wine samples (Stage 5) from the two wine-growing regions after spontaneous fermentation were detected by GC-MS. A total of 63 volatile compounds were identified in these samples (Supplementary Table S2), including 20 alcohols, 4 acids, 30 esters, 5 aldehydes and ketones, 2 hydrocarbons, 1 phenol, and 1 ether. Based on the types and concentrations of volatile compounds, PCA was performed on the wine samples (Figure 3A), and the cumulative variance contribution of the first two principal components reached 73.16%. The wine samples were distinguished based on their geographical origin, indicating significant differences in volatile compounds between the wine-growing regions (PERMANOVA, R^2^ = 0.67841, *p* = 0.001). The differences in the content of aromatic compounds in the wines were visualized through a heatmap (Figure 3B). In the wines from BC, higher concentrations were found for nine alcohols (1-octanol, 2-nonanol, 1-pentanol, etc.); two acids (octanoic acid and decanoic acid); four esters (ethyl butyrate, methyl salicylate, isobutyl decanoate, etc.); four aldehyde-ketone compounds (β-damascenone, α-ionone, 1-nonanal, etc.); and two hydrocarbons (1,3,5,7-cyclooctatetraene and 1,1,6-trimethyl-1,2-dihydronaphthalene). In contrast, the wine samples from NC exhibited higher levels of eleven alcohols (such as phenethyl alcohol, 3-methyl-1-butanol, and 1-butanol); two acids (hexanoic acid and benzoic acid); twenty-six esters (including isoamyl acetate, ethyl caprate, and ethyl caprylate); benzaldehyde; phenol; and phenetole.

### 3.4. Wines Fermented by Microorganisms with Different Geographical Origins Had Distinctive Aroma Profiles

**Figure 4:**
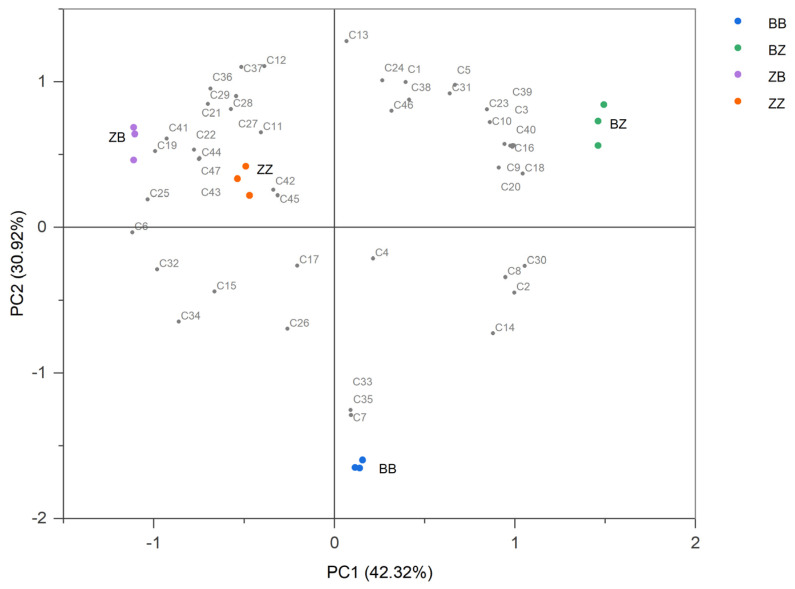
PCA biplot of volatile compounds in inoculated fermented wines. Note: volatile compound codes are provided in Table S3.

The impact of microbial communities on the aromatic profile of the wines was explored through inoculated fermentation. GC-MS was employed to detect the volatile compounds in the fermented wines inoculated with microbial communities from different geographical origins. A total of 47 volatile compounds were detected in these samples (Supplementary Table S3), including 16 alcohols, 24 esters, 2 aldehydes and ketones, and 5 acids. PCA was conducted on the aromatic substances in four groups of wines after inoculated fermentation to visualize the differences in aroma profiles among the samples (Figure 4). According to the differences in the types and concentrations of volatile compounds, the four groups of cabernet sauvignon wines were significantly distinguished (PERMANOVA, R^2^ = 0.93038, *p* = 0.001). The first principal component (PC1) contributed 42.32% of the variance, and the second principal component (PC2) contributed 30.92% of the variance, with the first two principal components explaining 73.24% of the total variance. The positive semi-axis region of PC1 included compounds such as C6 compounds (C4, hexyl alcohol; C18, hexanoic acid; C7, leaf alcohol) and D-citronellol (C14). The negative semi-axis region of PC1 contained esters such as ethyl butyrate (C45), ethyl caprylate (C27), and ethyl caproate (C32). Cabernet sauvignon wines were separated on the positive and negative semi-axes of PC1 based on the geographical origin of the grape juice. In the positive semi-axis region of PC2, volatile compounds that were strongly positively correlated with the sample’s score included phenylethyl alcohol (C1), ethyl caprylate (C27), and 2,3-butanediol (C12). The negative semi-axis region of PC2 included aromatic compounds such as leaf alcohol (C7), ethyl (E)-hex-2-enoate (C35), and ethyl glycolate (C33). The wines of BB were distributed in the fourth quadrant, and the remaining three groups of wines were clustered in the positive semi-axis region of PC2.

**Figure 5:**
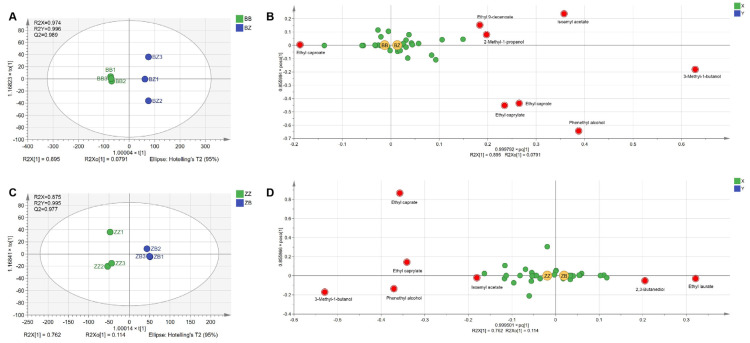
The volatile compounds in inoculated fermented wine varied depending on the geographical origin of the microbial community used for fermentation. OPLS-DA score scatter plot (**A**) and loading scatter plot (**B**) of volatile compounds in wines separately fermented by microbial communities from two regions in cabernet sauvignon juice from Beijing. OPLS-DA score scatter plot (**C**) and loading scatter plot (**D**) of aromatic compounds in wines separately fermented by microbial communities from two regions in cabernet sauvignon juice from Ningxia.

The results of the PCA provided preliminary evidence that the fermentation microbiota affected the final flavor of the wine. We further constructed an OPLS-DA model to identify key differential compounds and analyze the impact of microbiota from different geographical origins on the flavor of the wine. From the OPLS-DA results (Figure 5A,C), it can be observed that the wines fermented with microorganisms from the two different wine-growing regions were distinctly separated, indicating differences in their flavor compounds. Volatile compounds with VIP scores greater than 1 were the important contributors to the model. Microbial communities from the two regions were used to ferment cabernet sauvignon juice from B. The characteristic volatile compounds in the two groups of wines are shown in Figure 5B. 3-Methyl-1-butanol, phenylethyl alcohol, isoamyl acetate, ethyl caprate, ethyl caprylate, 2-methyl-1-propanol, and ethyl 9-decanoate could be identified as the characteristic volatile compounds of wines in BZ, while ethyl caproate could be identified as the characteristic volatile compound of wines in BB. Among the two groups of cabernet sauvignon juice from Z, 3-methyl-1-butanol, phenylethyl alcohol, isoamyl acetate, ethyl caprate, and ethyl caprylate were highly enriched in the wines of ZZ, while ethyl laurate and 2,3-butanediol were highly enriched in the wines of ZB (Figure 5D). These seven volatile compounds were the key compounds that distinguished the volatile profiles of these two groups of wines. Among them, 3-methyl-1-butanol, phenethyl alcohol, isoamyl acetate, ethyl caprate, and ethyl caprylate were the common characteristic volatile compounds in both BZ and ZZ, which may be related to the fermentation with the microbial community originating from Z. The results indicated that the fermentation microorganisms had an impact on the flavor characteristics of the wine, altering its overall aromatic quality.

### 3.5. Fungal Communities Associated with Wine Aroma Profiles

**Figure 6:**
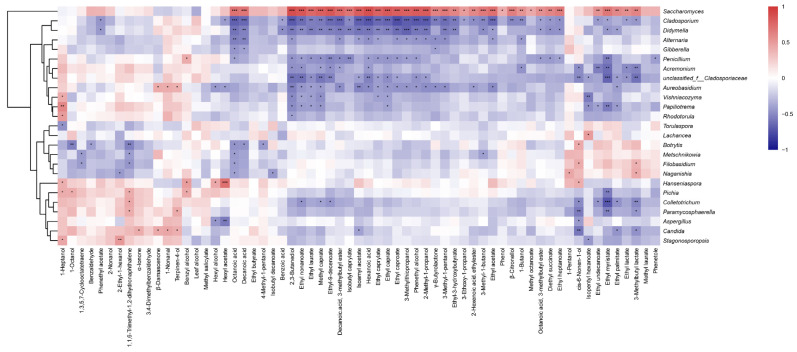
Relationship between fungal communities and aromatic compounds. Note: * 0.01 ≤ *p* < 0.05, ** 0.001 ≤ *p* < 0.01, *** *p* < 0.001.

To further elucidate the relationship between fungal microbiota and volatile metabolites in the wines, Spearman’s correlation analysis was used to explore the potential associations between fungal taxa and the synthesis metabolism of aroma compounds during fermentation. A heatmap of the correlation coefficients between fungal microbiota (relative abundance > 1% across samples) and volatile compounds was created (Figure 6). Fermentative yeasts are the main factor affecting the flavor and quality of wine, acting as the predominant producers of higher alcohols and esters [30]. In this study, it was observed that fermentative yeasts showed significant positive correlations with most volatile compounds (*p* < 0.05), while filamentous fungi such as *Cladosporium*, *Aureobasidium*, and *Alternaria* exhibited significant negative correlations with most volatile compounds (*p* < 0.05). For instance, *Saccharomyces* was positively correlated with esters, especially with some ethyl esters such as ethyl acetate, ethyl hexanoate, ethyl caprylate, and ethyl heptanoate, while fungal genera like *Cladosporium*, *Ddymella*, *Penicillium*, and *Aureobasidium* displayed significant negative correlations with these esters. *Candida* exhibited significant positive correlations with α-ionone, β-damascenone, terpinen-4-ol, and 1-nonanal but showed significant negative correlations with isoamyl acetate, cis-6-nonen-1-ol, ethyl palmitate, and 3-methylbutyl lactate. *Hanseniaspora* was significantly positively correlated with some alcohols (1-heptanol, benzyl alcohol, hexyl alcohol, and cis-6-nonen-1-ol) and hexyl acetate. *Torulaspora* exhibited a significant negative correlation with 1-heptanol, while *Lachancea* showed a significant positive correlation with isopentyl hexanoate. However, it is worth noting that the impact of microbiota on wine aroma may not necessarily be due to the direct production of specific volatile compounds, but rather the indirect regulation of the synthesis and degradation of these compounds in wine, affecting the overall metabolic activities of the microbial community [31].

## 4. Discussion

### 4.1. Biogeographical Distribution of Fungal Microbiota during Spontaneous Fermentation

Previous studies have shown that microbial communities associated with grapes and wines exist in a non-random geographical distribution pattern [13,32,33]. The fungal communities present on the skin of grape berries or in the musts from different wine-growing regions form unique fingerprint patterns that influence the production of volatile compounds in wines [34]. For example, on the surface of cabernet sauvignon berries harvested in the Washington State vineyards of the United States, the predominant fungal genera are *Cladosporium*, *Alternaria*, *Ulocladium*, and *Stemphylium* [35]. In cabernet sauvignon musts collected from the Griffith and Orange wine regions in Australia, *Aureobasidium* and *Mycosphaerella* were identified as the predominant genera within the fungal communities [4]. In this study, we collected samples from two wine-growing regions (880 km apart) that differed in geographical location, climatic conditions, and soil properties [20,21]. The main fungal genera in the musts from BC were *Cladosporium*, *Alternaria*, and *Colletotrichum*, whereas the most abundant genera in the musts from NC were *Cladosporium*, *Hanseniapora*, and *Saccharomyces*. There were significant differences in the fungal community composition and α-diversity between the two wine-growing regions, indicating distinct biogeographical patterns for fungal communities. At a large spatial scale, dispersal limitation may be a factor that promotes the formation of regional characteristics among microbial communities [36]. The geographical distribution patterns of microbial communities on grape surfaces and in musts can influence the profile of aromatic compounds in wine, enhancing the unique regional characteristics of the wine [18].

The structure of the microbial community varied with the fermentation stage. The early stage of alcoholic fermentation in wine is mainly initiated by non-*Saccharomyces* yeasts (mostly Crabtree-negative) such as those of the genera *Hanseniaspora*, *Metschnikowia*, and *Pichia*, which can quickly utilize glucose in musts. They mostly adhere to the surface of grape berries and are incorporated into the fermentation process along with the grape berries or juice [37]. As the alcoholic fermentation of wine proceeds, the increase in temperature and ethanol concentration in the fermentation environment, along with the low levels of available oxygen and the weak fermentation ability of non-*Saccharomyces* yeasts themselves [38,39,40], lead to a gradual decrease in the abundance of non-*Saccharomyces* yeasts (such as *Cladosporium*, *Alternaria*, and *Hanseniaspora*). At the same time, *Saccharomyces* gradually takes over and dominates the alcoholic fermentation process. The microbial structure of the fermentation is reshaped, and the diversity and abundance of microorganisms are decreased. However, in this study, the fungal community structure in the samples still exhibited differences according to their geographical origins at each fermentation stage. This indicated that the fungal communities retained their regional characteristics throughout the entire fermentation process.

### 4.2. Influence of Fungal Microbiota on Volatile Compounds in Wine

Aroma is one of the most important criteria for evaluating the quality of wine, and it is also a key parameter that reflects the style of the wine-growing region. The aroma compounds in wine are primarily divided into two categories. The first category consists of terpenes, methoxypyrazine, C6 compounds, etc., which play a predominant role in grape juice and the early fermentation period. The second category encompasses the major aromatic compounds in wine, including higher alcohols, esters, and volatile fatty acids. These compounds are primarily derived from the fermentation process of the wine and play a crucial role in contributing to its aromatic characteristics and sensory perception [29,41,42]. Macro-environmental factors like the climate and soil substrate are inherent elements that affect the flavor and quality of wine. Natural elements like the sunlight duration, precipitation, and soil conditions within the vineyard profoundly impact the growth and basic composition of wine grapes, thereby affecting the quality of the resulting wine [10]. However, wine fermentation is a complex process involving filamentous fungi, yeast, and bacteria. The interactions between these components begin in the vineyard and continue through to the packaging stage of the finished wine, jointly shaping the flavor characteristics of the wine. The microorganisms associated with winemaking are acquired conditions that influence the final flavor profile of the wine [43]. We also demonstrated this through fermentation experiments involving inoculation with microbiota from two different wine-growing regions. Our results showed that after fermenting the same cabernet sauvignon juice with microbiota from different wine-growing regions, there were differences in the volatile compounds present in the resulting wine after fermentation. This suggested that the diversity of microbial communities in musts can manifest in differences in the wine aroma compounds, ultimately altering the overall aroma profiles of wines. Microorganisms are an important factor influencing the flavor of wine.

Prior studies have suggested that the fungal communities associated with wine exhibit distinct distribution patterns at the regional scale, which can influence the flavor and quality of wine, contributing to the enhanced expression of flavor characteristics in wine-growing regions [5,11]. In this study, we analyzed the fungal community structure and concentration of aromatic compounds in wine samples. Our results were consistent with existing research, showing regional variations in the wine samples. These differences were observed in both the fungal community structure and the content of aromatic compounds in the wines. Yeasts are important microorganisms in wine production, and their metabolic activities and fermentation behavior greatly influence the chemical composition of wine [23]. Among them, *S. cerevisiae* plays a dominant role in the alcoholic fermentation of wine, serving as the primary producer of esters and higher alcohols and exerting a dominant influence on the flavor profile [11]. Non-*Saccharomyces* yeasts dominate the early stages of alcoholic fermentation in wine production. As the fermentation environment changes, they are gradually replaced by *S. cerevisiae*. Some non-*Saccharomyces* yeasts can secrete glycosidases, which have the ability to hydrolyze the odorless glycosidic precursors of volatile compounds (such as geraniol, phenethyl alcohol, nerol, and α-terpineol) into free aromatic substances, thereby increasing the complexity of the wine flavor [44,45]. Additionally, certain non-*Saccharomyces* yeasts have been shown to influence the final flavor profile of wine through different inoculation strategies during fermentation. For example, when *Rhodotorula mucilaginosa* was co-inoculated with *S. cerevisiae* for the fermentation of Ecolly dry white wine, there was an increase in the contents of the main aromatic compounds such as medium-chain fatty acids, ethyl acetate, ethyl butyrate, benzyl alcohol, and 1-hexanol, enhancing the wine’s floral and fruity characteristics [46]. The sequential inoculation of *Hanseniaspora vineae* followed by *S. cerevisiae* in Vidal blanc ice wine produced high levels of volatile compounds like (Z)-3-hexen-1-ol, (E)-3-hexen-1-ol, isobutanol, isoamyl acetate, cis-rose oxide, β-damascenone, and phenylacetaldehyde, resulting in heightened fruity, floral, and sweet sensory attributes [47]. The co-fermentation of *Issatchenkia terricola* and *Metschnikowia pulcherrima* with *S. cerevisiae* in cabernet sauvignon wine led to higher concentrations of C6 compounds, benzene derivatives, higher alcohols, and fatty acids, contributing an unpleasant green flavor to the wine [48]. The impact on the volatile compound content and flavor of wine depends on the species and individual characteristics of the strain [40]. Here, our results showed that *S. cerevisiae* had significant positive correlations with most esters, alcohols, and acids in the wine. Additionally, *Hanseniaspora*, *Metschnikowia*, *Torulaspora*, *Lachancea*, *Pichia*, and *Candida* showed positive correlations with certain alcohols, esters, and acids in the wine. The formation of wine flavor characteristics is not associated with a single microorganism but is the result of the participation of multiple microorganisms with complex interactions.

## 5. Conclusions

This study described the diversity of fungal communities during the spontaneous fermentation of cabernet sauvignon from two wine-growing regions in China and the impact of fungal communities on the aroma characteristics of the wines. The fungal compositions of cabernet sauvignon musts from different wine-growing regions, as well as throughout the entire fermentation process, exhibited significant differences, showing distinct regional characteristics. The biogeographical distribution patterns of the microbial communities in the musts were translated into aromatic differences in the wine. Fungal genera were correlated with different volatile compounds in the wine, and multiple microorganisms collectively affected the aromatic composition of the wine. A better understanding of the fungal communities in wine-growing regions and their influence on the volatile metabolites in wine would contribute to enhancing the expression of aroma profiles in wine-producing regions.

## Figures and Tables

**Table 1:** Physicochemical parameters in cabernet sauvignon musts and wines.

Region	Winery	Cabernet Sauvignon Musts (Must)	Cabernet Sauvignon Wines (Stage 5)				
		Glucose (g/L)	Fructose (g/L)	Glucose (g/L)	Fructose (g/L)	Glycerol (g/L)	Ethanol (% *v*/*v*)
BC	BLB	127.13 ± 0.21	169.89 ± 0.25	1.58 ± 0.26	0.50 ± 0.09	11.03 ± 0.12	16.56 ± 0.37
	XL	112.58 ± 0.40	149.50 ± 0.91	1.27 ± 0.13	1.18 ± 0.06	9.88 ± 0.13	14.54 ± 0.07
	ZW	116.07 ± 1.04	151.80 ± 1.47	0.23 ± 0.06	3.00 ± 0.33	8.34 ± 0.10	14.33 ± 0.07
NC	HJZ	140.42 ± 1.12	193.19 ± 1.50	1.04 ± 0.56	2.29 ± 0.16	12.00 ± 0.15	16.87 ± 0.06
	JX	140.51 ± 0.16	177.16 ± 0.35	2.05 ± 0.22	1.17 ± 0.24	9.07 ± 0.07	17.13 ± 0.33
	ZHYS	146.93 ± 1.83	186.05 ± 2.37	0.25 ± 0.08	1.53 ± 0.28	9.84 ± 0.18	18.87 ± 0.02

**Table 2:** Description of the abbreviations used for the inoculated fermentation samples.

Sample	Origin of Cabernet Sauvignon Juice	Origin of Microorganisms
BB	B	B
BZ	B	Z
ZZ	Z	Z
ZB	Z	B

## Data Availability

Data are contained within the article and Supplementary Materials.

## Supplementary Materials

The following supporting information can be downloaded at: https://www.mdpi.com/article/10.3390/foods13010106/s1, Figure S1: Sampling map of cabernet sauvignon from two wine-growing regions in China, located 880 km apart; Figure S2: The fungal genera with significant differences in relative abundance between two wine-growing regions at different fermentation stages; Table S1: Analysis of similarities (ANOSIM) using distance matrices of category effects on fungal communities in spontaneous fermentation; Table S2: Concentrations of volatile compounds in spontaneously fermented wines from two regions; Table S3: Concentrations of volatile compounds in inoculated fermented wines.
