# Supplementary material for: Fungal Community Composition and Its Relationship with Volatile Compounds during Spontaneous Fermentation of Cabernet Sauvignon from Two Chinese Wine-Growing Regions
Source: Foods. 2023 Dec 28;13(1):106. doi: 10.3390/foods13010106 (PMC10778937; doi:10.3390/foods13010106)
Supplement: Supplementary file 1 [file foods-13-00106-s001.zip › supplementary-Figures.pdf]

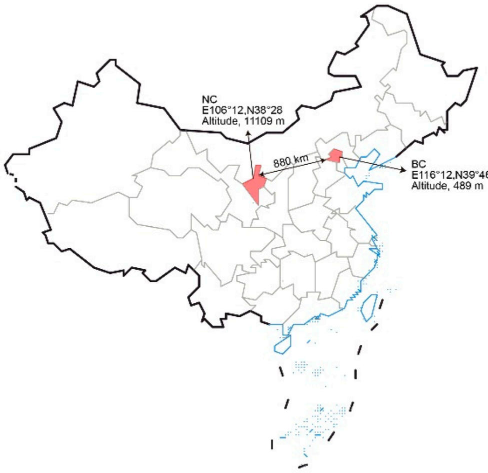

Figure S1. Sampling map of Cabernet Sauvignon from two wine-growing regions in China, located 880 kilometers apart.

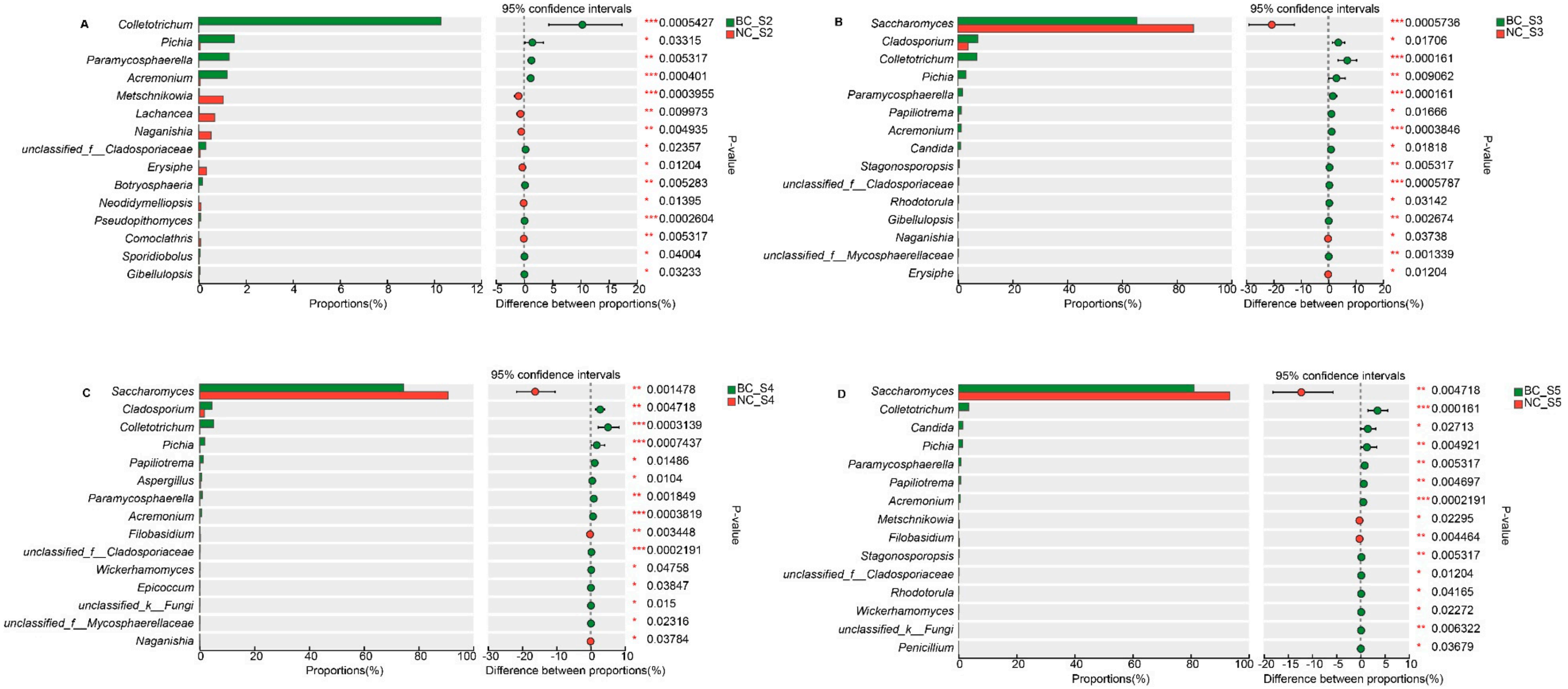

Figure S2. The fungal genera with significant differences in relative abundances between two wine-growing regions at different fermentation stages. Note: Wilcoxon rank-sum test, $p < 0.05$.
